# Women’s preference for a vaginal birth in Brazilian private hospitals: effects of a quality improvement project

**DOI:** 10.1186/s12978-024-01771-8

**Published:** 2024-03-28

**Authors:** Rosa Maria Soares Madeira Domingues, Marcos Augusto Bastos Dias, Maria do Carmo Leal

**Affiliations:** 1 Instituto Nacional de Infectologia Evandro Chagas/Fundação Oswaldo Cruz, Laboratório de Pesquisa Clínica em DST/Aids, Av. Brasil, 4365, Manguinhos, Rio de Janeiro, CEP 21040-360 Brazil; 2 Instituto Nacional da Saúde da Mulher, da Criança e do Adolescente Fernandes Figueira/Fundação Oswaldo Cruz, Rio de Janeiro, Brazil; 3 Escola Nacional de Saúde Pública Sérgio Arouca/ Fundação Oswaldo Cruz, Rio de Janeiro, Brazil

**Keywords:** Cesarean section, Childbirth, Patient preference, Quality improvement, Hospitals, Private, Brazil

## Abstract

**Background:**

In 2015, a quality improvement project called “Adequate Childbirth Project” (PPA) was implemented in Brazilian private hospitals in order to reduce cesarean sections without clinical indication. The PPA is structured in four components, one of which is directed at women and families. The objective of this study is to evaluate the effects of PPA on women’s preference for vaginal birth (VB) at the end of pregnancy.

**Methods:**

Evaluative research conducted in 12 private hospitals participating in the PPA. Interviews were carried out in the immediate postpartum period and medical record data were collected at hospital discharge. The implementation of PPA activities and women’s preference for type of birth at the beginning and end of pregnancy were compared in women assisted in the PPA model of care and in the standard of care model, using a chi-square statistical test. To estimate the effect of PPA on women’s preference for VB at the end of pregnancy, multiple logistic regression was performed with selection of variables using a causal diagram.

**Results:**

Four thousand seven hundred ninety-eight women were interviewed. The implementation of the planned activities of PPA was less than 50%, but were significantly more frequent among women assisted in the PPA model of care. Women in this group also showed a greater preference for VB at the beginning and end of pregnancy. The PPA showed an association with greater preference for VB at the end of pregnancy in primiparous (OR 2.54 95% CI 1.99–3.24) and multiparous women (OR 1.44 95% CI 0.97–2.12), although in multiparous this association was not significant. The main factor associated with the preference for VB at the end of pregnancy was the preference for this type of birth at the beginning of pregnancy, both in primiparous (OR 18.67 95% CI 14.22–24.50) and in multiparous women (OR 53.11 95% CI 37.31–75.60).

**Conclusions:**

The PPA had a positive effect on women’s preference for VB at the end of pregnancy. It is plausible that more intense effects are observed with the expansion of the implementation of the planned activities. Special attention should be given to information on the benefits of VB in early pregnancy.

**Supplementary Information:**

The online version contains supplementary material available at 10.1186/s12978-024-01771-8.

## Abstract (plain language summary)

Cesarean rates have been increasing worldwide and constitute the most frequent type of childbirth in Brazil since 2009. In 2015, a quality improvement project was implemented in Brazilian private hospitals, with the objective of reducing medically unnecessary cesarean sections and increasing the number of vaginal births. This project, called “Adequate Childbirth Project” (PPA), has four components, one of which is directed at women and families, aiming to increase their participation in decision-making processes related to childbirth. In this study, we assessed whether this program contributed to increased preference for vaginal birth at the end of pregnancy. In previous studies in Brazil, we saw that women who maintained preference for vaginal birth throughout pregnancy were those who had the lowest proportion of cesarean sections. We found that the PPA increased preference for vaginal birth by almost three times in primiparous women. Among women with previous births, this increase was smaller. In this group of women, having a previous cesarean section was an important factor for not wanting a vaginal birth, and this is a very common condition in Brazil. For all women, having preference for vaginal birth at the beginning of pregnancy was the main factor in wanting this type of birth at the end of pregnancy. The results demonstrate the importance of educational activities that disseminate information about the benefits of vaginal birth, increasing the preference of women for this type of childbirth, in addition to supporting them throughout pregnancy, so that they feel empowered in their choice.

**Supplementary Information:**

The online version contains supplementary material available at 10.1186/s12978-024-01771-8.

## Background

Since 2009, cesarean section (CS) represents the main type of childbirth in Brazil, accounting for 57.2% of live births in 2020 [1]. However, the distribution of these surgeries is uneven, being more frequent in women with more years of schooling and from higher economic class and assisted in private hospitals [2–4], a result also observed in other low, middle and high-income countries [5]. In Brazilian private hospitals, the childbirth model of care is centred on the obstetrician, usually the same one that provides antenatal care, with low participation of nurse-midwives [6] and high use of obstetric interventions [7, 8].

One of the explanations for the increase in CS in Brazil and other countries is the woman´s request, that is, a cesarean section requested from women without a medical indication. However, a systematic review has shown that only 15.6% of women prefer a CS at the beginning of pregnancy, reducing to 10.1% when excluding women with previous CS [9]. In Brazil, the “Birth in Brazil” study, a nationwide survey, conducted between 2011 and 2012, in public and private hospitals, showed an increase in the preference of women for CS [10], when compared to studies carried out in the 1990s [11]. However, only 27.6% of women reported preference for CS section at the beginning of pregnancy, with great variation according to parity and type of hospital during childbirth care. In primiparous women attended in public hospitals, the preference for CS at the beginning of pregnancy was 15.4%, reaching 73.2% in multiparous women with previous CS attended in the private sector [10].

The National Supplementary Health Agency (ANS), responsible for regulating the private health sector in the country, has adopted several initiatives to reduce CS in private hospitals. One of these initiatives is the “Adequate Childbirth Project” (“Projeto Parto Adequado”-PPA), developed in partnership with the Institute for Health Improvement and Hospital Israelita Albert Einstein, and supported by the Brazilian Ministry of Health. This is a quality improvement project, which aims to identify innovative and viable childbirth models of care that value vaginal birth and reduce the rate of CS without clinical indication in public and private health services. The PPA is structured in four components: governance, women’s empowerment, reorganization of the model of care and monitoring [12, 13].

The involvement of women in the formulation and implementation of childbirth models of care based on their needs, seeking a positive experience of childbirth, has been the focus of recent publications [14–17]. Interventions aimed at women and their families are among the non-clinical interventions for the reduction of medically unnecessary cesarean sections [17]. The PPA component directed at women and families aims to increase the participation of pregnant women and their families in the birth care process, through actions such as educational activities and campaigns, disseminating information about the project, visits to participating hospitals, and the development of a birth plan [12, 13].

The objectives of this study are i) to describe the decision-making process for type of birth in women assisted in Brazilian private hospitals participating in the PPA and ii) to evaluate the implementation of the activities of this project directed at women and their effects on the preference for vaginal birth (VB) at the end of pregnancy.

## Methods

### Study design

This is a cross-sectional evaluative study, called the “Healthy Birth” study, conducted in private hospitals that participated in the PPA.

The PPA is an ongoing quality improvement project of childbirth care, started in May 2015, and implemented in Brazilian public and private hospitals [12, 13]. It is structured in four theoretical components (Governance, Women and families, Reorganization of care, and Monitoring) with activities based on scientific evidence [18] and on 2 successful strategies for reducing caesarean sections in Brazilian private hospitals [8, 19].

At the hospital level, the project implemented new forms of care organization, with changes in the hospital environment, access to non-pharmacological methods for pain relief and equipment for births in vertical positions. Hospital staff included nurse-midwives in childbirth care and trained professionals implemented clinical guidelines. Activities for women included providing access to information, participation in educational groups, encouragement to develop a birth plan and visit to the hospital.

Each hospital participating in the PPA defined the population of women who would be the target of the project. In most of the hospitals the target population of the PPA consisted of women admitted by the hospital’s on-call staff, while others included all primiparous or women in Robson groups 1 to 4 [20]. Women not targeted by the PPA were assisted in the standard of care model of Brazilian private hospitals which is characterized by women usually seen by the same doctor during prenatal and childbirth care [8], low participation of nurse-midwives [6], a high proportion of antepartum cesarean section [10], low use of labor induction [10], low use of best practices and high use of obstetric interventions during labor [7]. More information about the PPA is available at the ANS website [12] and at Boren et al. [13].

In 2017, an evaluative research called the “Healthy Birth” study was carried out to assess the degree of implementation and the effects of the PPA in a convenience sample of 12 hospitals, among the 23 private hospitals that participated in the first phase of the project [21]. For each maternity hospital, a sample size of 400 women was calculated, aiming to detect a 10% reduction in the proportion of CS, considering an estimate of 50%, 80% power and 5% significance level.

All women with live births of any weight or gestational age or stillbirths weighing 500 g or more or with 22 or more gestational weeks were eligible for the “Healthy Birth” study. Exclusions included home births or public deliveries, women with legal determination to terminate pregnancy, women with triplets pregnancy or more, and women who did not speak Portuguese or with hearing and speaking impairments, due to the difficulties in conducting the face-to-face interviews.

All women admitted for childbirth care in the 12 hospitals, assisted in the “PPA model of care” or in the “standard of care model”, and who met the eligibility criteria were invited to participate in the “Healthy Birth” study, until 400 participants were included in each hospital. We carried out face-to-face interviews with women at least 6 h after birth and extracted data from their prenatal card. After hospital discharge, we extracted data from the medical records of the woman and newborn. All instruments used for data collection were developed for the “Healthy Birth” study and are available at Torres et al. [21].

In the present analysis, we: i) describe the implementation of activities provided for in the PPA component directed at women, ii) describe the decision-making process of women from the preference at the beginning of pregnancy through the actual type of birth, and iii) estimate the effect of PPA on women’s preference for the type of birth at the end of pregnancy.

Throughout the analyses, we compared the two models of care: the “PPA model of care”, based on the recommendations of the PPA quality improvement project, and “the standard of care model”, adopted routinely in private services. For the classification of women according to the model of care (PPA or standard of care), we used data from the interview with the women (to identify the team responsible for the care) and hospital records (for obstetric characteristics and Robson’s group).

First, we compared the demographic, social and obstetric characteristics of women assisted in the two models of care. In the economic classification, women in class “A” represent those of the highest economic level [22]. For parity classification, we considered primiparous those who were having their first birth and multiparous those who had a previous birth, regardless of the number. We classified women presenting any of the following conditions that could affect their preference for CS as having complications during pregnancy: hypertensive syndromes, gestational and non-gestational diabetes, placenta previa, infections (HIV, Zika virus), congenital malformation, intrauterine growth restriction, oligodramnia and polydramnia.

We described and compared the implementation of activities planned in the PPA component directed at women—which included providing information about the PPA, offering visits to maternity hospitals, offering educational activities in antenatal groups, counseling to prepare a birth plan and providing information on various aspects of labor and childbirth care—between women assisted in the two models of care.

We assessed and compared the preference for type of birth in early pregnancy and at the end of pregnancy and the reasons for this preference or changes in the preference among primiparous and multiparous assisted in the two models of care. The preference for the type of birth in early pregnancy and at the end of pregnancy was assessed retrospectively, during the face-to-face interviews carried out with women in the immediate postpartum. For women with a preference for a type of birth at the beginning of pregnancy, we described the decision process, according to parity and model of care, from the initial preference to the actual type of birth. In all comparative analyzes between the PPA model of care and the standard of care model, we used the chi-square statistical test with a significance level of 5% to detect differences between proportions.

Finally, we conducted a multiple logistic regression to estimate the effect of PPA on the preference for VB at the end of pregnancy, using a causal diagram [23] for the selection of the minimum set of variables for adjustment. We used two different causal diagrams, one for primiparous and the other for multiparous, available in the (Supplementary material: Figs. 1 and 2). In primiparous women, the selected variables were type of pregnancy (single or multiple with two fetuses), fetal presentation (cephalic or breech), duration of pregnancy (term or preterm), pregnancy complications (yes, no) and economic class (A, B, C, D/E). For multiparous women, the model also included the variable “previous cesarean section” (yes / no).

All statistical analyses were performed using SPSS software version 17 (https://www.Ibm.com/), using data weighting and incorporating the design effect, considering the complex sampling process.

## Results

We interviewed 4798 women, 53.6% assisted in the PPA model of care and 46.4% in the standard of care model. Most women declared themselves white, had 15 or more years of schooling, belonged to the higher economic classes “A” and “B”, lived with their partner, had paid work and were primiparous. Among those with previous birth, 73.4% had a previous cesarean section. The largest proportion of women belonged to Robson’s groups 2b and 5, which correspond respectively to primiparous women with single pregnancies, cephalic, full term, with antepartum cesarean section and multiparous women with single, cephalic and full-term pregnancies with previous CS. A quarter of women had clinical and/or obstetric complications that could affect their preference for the type of birth at the end of pregnancy. Women in the standard of care model were older, belonged to higher economic classes, more frequently lived with their partners, were more frequently multiparous, and had a higher proportion of previous CS and complications during pregnancy, while more women in the PPA model of care had paid work. Almost half of the women assisted in the standard of care model belonged to Robson’s group 5, while in the PPA model of care the most frequent groups were groups 2b and 1 (Table 1).

**Table 1:** Demographic, social and obstetric characteristics of women assisted in the two models of care

Maternal Characteristics	Total (*N* = 4,798)		PPA model of care (*N* = 2,571)		Standard of care model (*N* = 2,227)		*P* value
	**n**	**%**	**n**	**%**	**N**	**%**	
Age							< 0.001
< 20	83	1.7	67	2.6	16	0.7	
20 to 34	3067	63.9	1753	68.2	1315	59.0	
35 or more	1647	34.3	750	29.2	897	40.3	
Self-reported skin color^a^							0.215
White	3239	67.6	1733	67.5	1506	67.7	
Black	229	4.8	136	5.3	94	4.2	
Mixed	1194	24.9	622	24.2	572	25.7	
Asian	129	2.7	77	3,0	52	2.3	
Years of schooling							0.563
1 to 10	204	4.3	117	4.6	87	3.9	
11 to 14	1859	38.9	997	39,0	862	38.8	
15 or more	1414	29.6	738	28.9	677	30.5	
Post-graduate studies	1298	27.2	702	27.5	596	26.8	
Economic class							< 0.001
D/E	14	0.3	9	0.4	5	0.2	
C	797	16.6	473	18.4	324	14.6	
B	2614	54.5	1414	55.0	1200	53.9	
A	1373	28.6	674	26.2	698	31.4	
Lives with partner	4475	93.7	2352	92.0	2123	95.6	< 0.001
Paid work	3793	79.4	2075	81.2	1718	77.3	0.004
Primiparous	2846	59.4	2084	81.1	762	34.3	< 0.001
Previous cesarean section^b^	1428	73.4	182	37.3	1246	85.4	< 0.001
Robson´s groups							< 0.001
1.Nulliparous, single, term, cephalic, spontaneous labour	817	17.0	718	27.9	99	4.4	
2a. Nulliparous, single, ≥ 37 ws, cepahlic, induced labour	270	5.6	252	9.8	18	0.8	
2b. Nulliparous, single, ≥ 37 ws, cephalic, CS before labour	1233	25.7	895	34.8	338	15.2	
3. Multiparous, single, ≥ 37 ws, cephalic, spontaneous labour	250	5.2	182	7,1	68	3,1	
4a. Multiparous, single, ≥ 37 ws, cepahlic, induced labour	63	1.3	50	1.9	13	0.6	
4b. Multiparous, single, ≥ 37 ws, cephalic, CS before labour	127	2.6	52	2,0	75	3.4	
5. Previous CS, single, cephalic, ≥ 37 weeks	1219	25,4	146	5.7	1072	48.2	
6. All nullipara breeches	187	3.9	80	3.1	107	4.8	
7. All multípara breeches (including previous CS)	93	1.9	16	0.6	77	3.5	
8. All multiple pregnancies (including previous CS)	84	1.8	19	0.7	65	2.9	
9. All abnormal lies (including previou CS)	39	0.8	6	0.2	33	1.5	
10. All single cephalic ≤ 36 weeks (including previous CS)	418	8.7	156	6,1	262	11.7	
Complications during pregnancy	1199	25.0	594	23.2	605	27.2	0.005

*Ws* Weeks, *CS* Cesarean section

^a^Classification of skin color used in the Brazilian demographic census

^b^Among women with previous birth (*n* = 1948)

Of the planned activities in the PPA directed at women, the “orientation to visit the maternity hospital” was the most frequent. Receipt of information about the PPA, invitation to participate and participation in antenatal groups of pregnant women and counseling to prepare a birth plan were reported by less than a third of women. Less than 10% of women prepared a birth plan. Access to information was high, as more than 70% of women reported having received information about various aspects of childbirth, except for delayed umbilical cord clamping, reported by 43% of women. At the end of pregnancy, 46.5% of women considered that VB was the safest type of birth for herself and for the baby, while 46.2% considered that VB and CS were equally safe. Women assisted in the PPA model of care more frequently participated in all planned activities, except being counseled to give birth in a PPA maternity hospital. They also received more information about signs of labor, best practices in childbirth care, delayed umbilical cord clamping, and considered VB to be the safest (Table 2).

**Table 2:** Access to health activities and health information according to the type of model of care

**Health activities**	**Total (*n* = 4,798)**		**PPA model of care (*n* = 2,571)**		**Standard of care model (*n* = 2,227)**		***P* value**
	**n**	**%**	**n**	**%**	**n**	**%**	
Received information about the PPA	1582	33.0	889	34.6	693	31.1	0.025
Counseled to give birth in a PPA hospital^a^	355	22.4	207	23.3	147	21.2	0.354
Opted to give birth in a PPA hospital^a^	589	37.2	385	43.3	204	29.4	< 0.001
Counseled to visit the maternity hospital	2523	52.6	1438	55.9	1084	48.7	< 0.001
Visited the maternity hospital	1620	33.8	1060	41.2	560	25.1	< 0.001
Offered to participate in an antenatal group activity	1582	33.0	917	35.7	665	29.9	0.001
Participated in the antenatal group activity^b^	427	27.0	272	29.7	154	23.2	0.014
Counseled to prepare a birth plan	697	14.5	428	16.7	269	12.1	< 0.001
Prepared a birth plan	467	9.7	300	11.7	167	7.5	< 0.001
Received information about:							
Signs of labor	4082	85.1	2245	87.3	1837	82.5	< 0.001
Signs of risk danger	4050	84.4	2193	85.3	1857	83.4	0.120
Best practices during labor ^c^	3652	76.1	2035	79.1	1618	72.6	< 0.001
Delayed umbilical cord clamping	2060	43.0	1153	44.9	907	40.7	0.014
Skin to skin contact immediately after birth	3454	72.0	1832	71.3	1621	72.8	0.297
Breastfeeding during the first hour after birth	3656	76.2	1957	76.1	1699	76.3	0.908
Risks and benefits of each type of birth	3464	72.3	1830	71.3	1634	73.4	0.158
Safest type of birth for mother and baby							< 0.001
Vaginal	2177	46.5	1262	50.4	915	42.1	
C section	338	7.2	121	4.8	218	8.8	
Both are equally safe	2163	46.2	1122	44.8	1041	47.9	

^a^Among those who received information about the PPA (*n* = 1582)

^b^Among those who were offered to participate in the group activities (*n* = 1582)

^c^Freedom of movement, bathing, adopting upright positions, non-pharmacological pain relief

Among primiparous women, approximately a quarter preferred a CS at the beginning of pregnancy, while in multiparous women this preference was reported by almost 50%. Significant differences were observed in the two models of care, with a greater preference for VB, both in primiparous and multiparous women, in the PPA model of care (Table 3). For all women, the most cited reason for the initial preference for VB was the benefits of this type of birth, especially its faster recovery, but also because it is more physiological and due to the birth experience. In primiparous women, information received and benefits for the baby were the other most cited factors, while in multiparous women, the previous positive experience with VB was reported by a quarter of the women. For the preference for CS, the reason most cited by primiparous women was fear of pain in childbirth, followed by stories of other women, health problems and convenience of the CS. In multiparous women, the previous positive experience with CS was the most frequent reason, followed by fear of pain in childbirth.

**Table 3:** Women´s preference of type of birth according to parity and type of model of care

**Women´s preferences**	**Primiparous women**							**Multiparous women**						
	**Total (*n* = 2,846)**		**PPA model of care (*n* = 2,084)**		**Standard of care model (*n* = 762)**		***P* value**	**Total (*n* = 1,948)**		**PPA model of care (*n* = 487)**		**Standard of care model (*n* = 1,461)**		***P* value**
	**n**	**%**	**n**	**%**	**n**	**%**		**n**	**%**	**n**	**%**	**n**	**%**	
Preference of type of birth in early pregnancy							< 0.001							< 0.001
Vaginal	1825	64.1	1428	68.5	397	52.1		911	46.8	340	69.8	572	39.1	
C-section	745	26.2	467	22.4	278	36.5		900	46.2	132	27.2	767	52.5	
No preference	275	9.7	188	9.0	87	11.4		137	7.0	15	3.0	122	8.4	
Reasons to prefer vaginal birth:														
Benefits of vaginal birth	1577	86.4	1247	87.4	329	83.0	0.054	671	73.7	244	71.9	427	74.7	0.421
Fear of C-section	116	6.3	99	7.0	16	4.1	0.083	46	5.1	22	6.5	24	4.2	0.221
Benefits for the child	180	9.9	149	10.4	31	7.9	0.155	58	6.3	18	5.3	40	7.0	0.443
Information	308	17.0	236	16.5	73	18.6	0.412	117	12.8	44	13.1	73	12.7	0.898
Other women´s histories	154	8.5	125	8.8	29	7.4	0.422	45	5.0	25	7.3	20	3.6	0.019
Previous positive experience with vaginal birth								234	25.7	137	40.3	98	17.0	< 0.001
Previous negative experience with C-section								67	7.3	13	3.7	54	9.5	0.005
Reasons to prefer C-section														
Fear of labor pain	424	56.8	270	57.9	153	55.1	0.512	272	30.2	38	28.5	234	30.5	0.616
Fear of vaginal birth	47	6.3	32	6.9	15	5.2	0.413	28	3.2	1	0.6	28	3.6	0.050
Information	35	4.7	22	4.7	14	4.9	0.902	25	2.7	5	3.9	20	2.5	0.333
Convenience	75	10.0	41	8.8	34	12.2	0.192	55	6.1	9	6.8	46	6.0	0.694
Safety	10	1.3	6	1.3	4	1.3	0.990	4	0.4	1	0.4	3	0.4	0.948
Benefits for the child	5	0.7	3	0.7	2	0.7	0.895	2	0.2	1	0.6	1	0.2	0.285
Woman´s health problems	85	11.4	47	10.0	38	13.6	0.207	81	9.0	11	8.4	70	9.1	0.826
Other women´s histories	114	15.3	76	16.2	39	13.9	0.428	42	4.7	8	6.1	34	4.5	0.409
Twin pregnancy	10	1.3	2	0.5	7	2.6	0.014	16	1.8	0		16	2.1	0.177
Tubal ligation								81	9.0	16	12.4	64	8.4	0.096
Previous C-section								78	8.6	19	14.6	58	7.6	0.008
Previous positive experience with C-section								345	38.4	37	28.2	308	40.2	0.005
Previous negative experience with vaginal birth								82	9.1	20	15.0	62	8.0	0.020
Changed preference during pregnancy														
Vaginal to C-section	326	17.9	204	14.3	122	30.8	< 0.001	193	21.2	46	13.7	146	25.6	< 0,001
C-section to Vaginal	156	21.0	123	26.4	33	11.8	< 0.001	45	5.0	12	9.3	32	4.2	0.005
Reasons for changing preference (vaginal to C-section)														
Woman´s health problem	101	30.8	65	32.1	35	28.7	0.512	53	27.4	13	29.1	39	26.9	0.799
Fetus condition	101	31.0	52	25.6	49	40.0	0.008	53	27.5	12	26.5	41	27.8	0.869
Information provided by obstetrician	30	9.3	16	7.9	14	11.7	0.293	35	17.9	8	16.4	27	18.4	0.801
Convenience of C-section	42	12.9	29	14.2	13	10.8	0.361	17	8.9	4	9.6	13	8.6	0.835
Fear of vaginal birth	76	23.3	59	29.1	17	13.5	0.001	32	16.5	7	15.2	25	16.9	0.833
Previous C-section								6	3.0	1	1.8	5	3.4	0.540
Tubal ligation								7	3.6	2	4.1	5	3.5	0.809
Reasons for changing preference (C-section to vaginal)														
Benefits of a vaginal birth	38	24.1	32	25.7	6	18.0	0.392	14	30.7	4	29.8	10	31.0	0.933
Family and friends	17	10.8	13	10.4	4	12.2	0.826	3	6.5	1	5.8	2	6.8	0.884
Fear of C-section	13	8.1	12	9.4	1	3.1	0.147	4	8.0	1	3.8	3	9.6	0.398
Information provided by obstetrician	52	33.2	44	35.8	8	23.4	0.207	16	35.2	4	32.5	12	36.2	0.788
Other sources of information	69	44.1	50	40.3	19	58.4	0.114	7	16.2	3	22.1	4	13.9	0.437
Preference of type of birth at the end of pregnancy							< 0.001							< 0.001
Vaginal	1655	58.1	1347	64.6	307	40.3		764	39.2	306	62.8	459	31.4	
C-section	914	32.1	547	26.2	368	48.2		1046	53.7	166	34.2	880	60.2	
Not informed	278	9.8	191	9.1	87	11.4		138	7.1	15	3.0	123	8.4	

The reasons for preference for the type of birth were similar in women assisted in the two models of care. Among multiparous women, a positive experience with the previous type of birth was frequently cited to prefer the same type of birth in the current pregnancy. However, the type of birth varied in the two models of care: in the standard of care model, women reported their previous experience with CS more frequently, while women in the PPA model of care cited their experience with a VB (Table 3).

Changes in preference for the type of birth were observed throughout pregnancy. Among primiparous women, about a fifth of women reported change of preference from VB to CS or from CS to VB. In multiparous women, the biggest change was from VB to CS, with only 5.0% changing the preference from CS to VB. Primiparous and multiparous women in the PPA model of care had a significantly greater change in preference from CS to VB, while primiparous and multiparous women in the standard of care model had a significantly greater change in preference from VB to CS (Table 3). The main reasons for changing the preference from VB to CS, both in primiparous and multiparous women, were women’s health problems and the fetus condition. The only significant difference between the two models of care was the fear of VB and the fetus condition, reported more frequently by primiparous women assisted in the PPA model of care. Among the reasons for changing preference from CS to VB, the most cited were the information received and the benefits of vaginal birth, with no significant differences in the two models of care. Multiparous women more often cited the obstetrician as a source of information, while primiparous women cited other sources of information. With changes in preference, at the end of pregnancy, 57.5% of primiparous and 38.7% of multiparous preferred a VB. Significant differences were observed in primiparous and multiparous women assisted in both models of care, with a significantly higher proportion of preference for VB at the end of pregnancy in women assisted in the PPA model of care (Table 3).

**Fig. 1:**
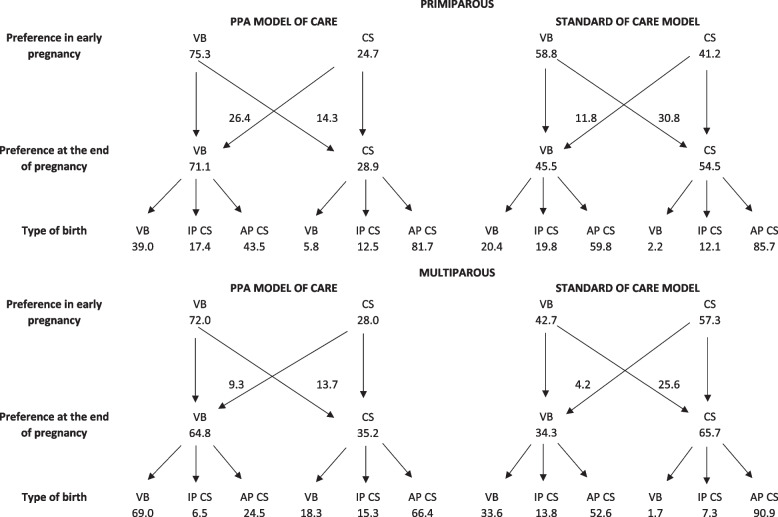
Decision-making process of the type of delivery according to parity and model of care. Legend: VB = vaginal birth; IP CS = intrapartum cesarean section; AP CS = antepartum cesarean section

In Fig. 1, the decision process is described for women who had a preference for the type of birth at the beginning of pregnancy. In the PPA model of care, 75.3% of primiparous and 72.0% of multiparous women preferred a VB at the beginning of pregnancy. At the end of pregnancy, this preference reduced to 71.1% and 64.8%, respectively, remaining as the most frequent. The highest proportion of VB was observed in women who preferred this type of birth at the end of pregnancy, especially in multiparous women, where 69% of women with a preference for VB had this type of birth. In women assisted in the standard of care model, the initial preference for CS was already greater in multiparous women at the beginning of pregnancy, increased at the end of pregnancy and resulted in 90.9% of antepartum CS in women with preference for this type of birth. In primiparous women assisted in the standard of care model, the preference for VB was greater at the beginning of pregnancy, but was reversed at the end of pregnancy, resulting in only 20.4% ​​of VB in those who maintained this preference.

The PPA model of care almost tripled the preference for VB at the end of pregnancy in primiparous women (OR 2.54 95% CI 1.99–3.24), but its effect was smaller in multiparous women (OR 1.44 95% CI 0.97–2.12). In both groups, the main predictor of preference for VB at the end of pregnancy was the preference for this type of birth at the beginning of pregnancy. In primiparous women, having a single pregnancy, having no complications during pregnancy and belonging to the highest economic classes “A” and “B” were also positively associated with the preference for VB at the end of pregnancy, while having a term birth was negatively associated with this preference. In multiparous women, the only factors associated with the preference for a VB at the end of pregnancy were the preference for VB in early pregnancy and not having a previous CS (Table 4).

**Table 4:** Multivariate logistic regression for vaginal birth preference at the end of pregnancy according to parity

**Maternal characteristics**	**Primiparous (*N* = 2,611)**				**Multiparous (*N* = 1,842)**			
	**%**	**ORa**	**95% CI**	***P* value**	**%**	**ORa**	**95% CI**	***P* value**
Model of care								
PPA	73.8	2.54	1.99–3.24	< 0.001	26.2	1.44	0.97–2.12	0.069
Standard of care	26.2	1			73.8	1		
Economic class								
A	24.9	2.29	1.59–3.30	< 0.001	32.1	1.06	0.64–1.76	0.926
B	58.1	1.36	1.01–1.83		49.8	1.09	0.70–1.70	
C/D/E	17.0	1			18.1	1		
Preference of type of birth in early pregnancy^a^								
Vaginal	71.1	18.67	14.22–24.50	< 0.001	50.5	53.11	37.31–75.60	< 0.001
Cesarean section	28.9	1			49.5	1		
Type of pregnancy								
Single	98.4	5.25	2.13–12.92	< 0.001	98.3	2.30	0.22–23.57	0.484
Multiple	1.6	1			1.7	1		
Fetal presentation								
Cephalic	92.5	1.09	0.67–1.78	0.727	93.9	0.77	0.43–1.37	0.374
Breech/others	7.5	1			6.1	1		
Gestational duration								
Term	88.5	0.61	0.43–0.88	0.008	90.3	1.34	0.74–2.41	0.331
Preterm	11.5	1			9.7	1		
Complications during pregnancy								
No	76.3	1.65	1.27–2.15	< 0.001	74.0	1.27	0.88–1.82	0.203
Yes	23.7	1			26.0	1		
Previous Cesarean section								
No					28.3	2.62	1.79–3.85	< 0.001
Yes	NA	NA	NA	NA	71.7	1		

*NA* Not applicable

^a^Women without preference of type of birth at early pregnancy were not included in this analysis

## Discussion

The results of this study show that the PPA had an effect on women’s preference for the type of birth at the end of pregnancy, especially in primiparous women, where the preference for VB was 2.5 times higher in women assisted in the PPA model of care, even after adjusting for potentially confounding factors. The lower number of multiparous women in the PPA model of care may have reduced the study’s power to detect the effect of PPA in multiparous women.

We observed a positive effect of PPA on women’s preference for VB even with the low implementation of planned activities. However, the women assisted in the PPA model of care reported greater participation in several activities, such as visiting the maternity-hospitals, participating in antenatal groups, preparing a birth plan and choosing to give birth in a hospital participating in the PPA, which may reflect women’s adherence to the proposed model of care. It is plausible to suppose that greater effects can be observed with the increase in the degree of implementation of activities, such as greater dissemination of the PPA objectives and activities and the provision of educational activities. Promoting vaginal birth, through health education activities, is a strategy for reducing medically unnecessary cesarean sections [24, 25].

There was an increase in preference for VB at the beginning of pregnancy, when compared to results observed in private hospitals between 2011 and 2012 [10], although in lower rates ​​than those observed in other countries [9, 24, 26–30]. However, this increase is probably due to the greater preference for VB reported by primiparous and multiparous women assisted in the PPA model of care. In the standard of care model, the preference for VB at the beginning of pregnancy in primiparous and multiparous women was similar to that found in 2011–2012 [10], showing that there was little change in the preference profile of women assisted in the standard of care model in Brazilian private hospitals.

Although there was a reduction in the preference for VB throughout pregnancy in all women, similarly to that observed in previous studies in Brazilian private hospitals [10, 31], the reduction was more pronounced in the standard of care model. Both primiparous and multiparous women assisted in the PPA model of care showed a greater change in preference from cesarean section to vaginal birth throughout pregnancy than women in the standard of care model.

In both primiparous and multiparous women, the main factor associated with the preference for VB at the end of pregnancy was the initial preference for this type of birth, with stronger effects in multiparous women. Similar to previous studies [10, 11, 27, 31–34], the main reason for the initial preference for a VB was the benefits of this type of birth. However, there was a 4 to 5-fold increase in the proportion of women who cited information as the reason for their preference for vaginal birth: 17% in primiparous women and 13% in multiparous women, when compared to the rate below 3% observed in 2011–2012 [10]. Health benefits to the baby were also cited by 10% of primiparous women as a reason for the preference for a VB. In a previous study, safety was cited only to justify the preference for CS [10], similarly to other countries [24, 25, 27, 29, 35–37]. In a systematic review conducted in China [29], there was evidence in studies published in more recent years that women’s and health professionals’ beliefs may be shifting towards valuing more the vaginal birth.

In women assisted in the PPA model of care, the greater receipt of information on signs of labor and best practices in labor and childbirth care, the more positive perception of the safety of vaginal birth for women and babies, and access to information as a reason for preference for vaginal birth, both at the beginning of pregnancy and as a motive to change preference during pregnancy, may reflect the greater commitment of health care providers to an evidence-based model of care. The values ​​and preferences of women in relation to the type of birth vary in populations and in the same woman over time, and face-to-face interaction with health professionals is the main influence in decision of the type of birth [38].

However, women sought information from sources other than the obstetrician, especially primiparous women, a result also observed in a systematic review of women´s request for an elective cesarean section for non-medical reasons [25]. In this study, belonging to a higher economic class was an independent factor for preference for VB at the end of pregnancy in primiparous women. It is possible that these women had greater access to information sources other than their obstetrician, reflecting a greater demand for different models of care by women who are experiencing their first birth. It is worth mentioning that the PPA is a response of ANS to a lawsuit brought by the women’s movement in the State of São Paulo, which required measures to curb the excess of CS observed in Brazilian private hospitals, reflecting dissatisfaction with the current model of care and a demand for new ones.

Our results point to several challenges. Among the reasons for the preference for CS, the fear of VB stands out, reported by more than half of primiparous women and nearly one third of multiparous women. This is the most frequently reason cited in the literature for the preference for CS [24–26, 28, 29, 35, 39, 40] and, in this study, a reason significantly more reported by primiparous women assisted in the PPA model of care to change preference for CS during pregnancy. In international studies, the prevalence of childbirth fear varies between 24%-26%, with studies reporting an association of childbirth fear with giving birth for the first time and previous operative birth (forceps, vacuum, elective or emergency CS) [41, 42]. In addition to fear of pain, fear of VB may be related to the expectations of the care that will be provided. Unsatisfactory relationships with health professionals and perceived deterioration in the quality of care during labor and childbirth were identified as reasons for preferring CS in China [29]. A recent global qualitative evidence synthesis concluded that a wide variety of factors underlie women’s preferences for CS in the absence of medical indications but that the major factors contributing to perceptions of CS as preferable include fear of pain, uncertainty with vaginal birth and positive views on CS [40].

The occurrence of complications during pregnancy was a factor associated with a lower preference for VB at the end of pregnancy in primiparous women. In fact, complications that are direct indications for CS do not allow a woman’s preference for a vaginal birth to be guaranteed. However, it is known that, in Brazil, there is an excess of cesarean sections determined by non-clinical factors [5]. It is possible that greater flexibility in the indications for CS in private services [4], associated with the fear of VB, the insecurity of the first birth and counseling received from the health care provider [30] affect the preference of primiparous women during pregnancy, which may also explain the lower preference for VB at the end of pregnancy in primiparous women at term observed in this study.

In multiparous women, having a previous CS was an independent factor for the lower preference for VB at the end of pregnancy. The greater preference of multiparous women for CS, especially in women with previous CS, was also observed in two systematic reviews of women´s preference for type of birth [9, 29]. This finding is particularly important in Brazil, where the proportion of CS in primiparous women is high and the rate of vaginal birth after a cesarean section (VBAC) is very low [10, 43]. One third of cesarean sections in Brazilian private hospitals [44, 45] occur in women with previous CS, a finding similar to that observed in developed countries, where approximately 30% of cesarean sections have a previous CS as their primary indication [46].

Several factors may affect the women´s preference for the type of birth after a previous CS, including previous reproductive experience, risk factors in the current pregnancy, personal preferences and social context [30, 46, 47]. In a study carried out in Switzerland, the counseling received on VBAC was the strongest predictor for the preference for VB in women with a previous CS [47]. However, the evidence on the best way to support women in this decision-making process is still limited [48, 49]. It is likely that in a context where the possibility of VB after a previous CS section is practically non-existent, as in Brazil, women’s preference for a VB is affected, reinforcing the belief that “once cesarean, always cesarean”, despite contrary evidence [50].

The women assisted in the PPA model of care were younger and mostly primiparous. This is a positive aspect of the program, as primiparous women must be the target of any strategy aimed at reducing CS, seeking to prevent primary cesarean sections. However, half of the hospitals did not include women with a previous CS, who represent more than a quarter of the women assisted in Brazilian private hospitals [44]. In this study, 34.9% of women with a previous CS preferred a VB at the beginning of their current pregnancy (data not shown), a result similar to that observed in the USA, where 45% of women at 12 months after a CS expressed a preference for VB in a future pregnancy [51]. These women could benefit from the actions of the PPA, with an expectation of increase in the proportion of VBAC in Brazilian private hospitals. Women with a previous CS who preferred a VB, but who had a repeat CS, had worse Health Related Quality of Life three months after birth, with unknown long-term outcomes, according to a recent study conducted in 15 maternity hospitals in three European countries [52]. Low values ​​of VBAC rates have been observed in developed countries, with increasing use of repeated cesarean sections being the factor that has most contributed to the high rates of cesarean sections in these countries [46].

Women who maintained the option for VB at the end of pregnancy were those who had the highest proportion of this type of birth. However, the proportion of VB was much lower than the preference reported by women in late pregnancy, a result also observed in previous studies [10, 29, 31]. The lower proportion of VB may reflect complications arising during labor, but also the performance of a CS for woman´s request during labor, due to pain, fear and suffering, especially in environments with little social support and availability of methods for pain relief [30]. Compared to 2011, there was an increase in the availability of non-pharmacological methods for pain relief and epidural analgesia in Brazilian private hospitals, but the use of interventions during labor and childbirth is high and the implementation of best practices is still low [53].

To reduce unnecessary cesarean sections, strategies directed at women must be linked to other interventions aimed at health professionals and the hospital environment, including social support, assistance by nurse-midwives/midwives, the training of health care providers and implementation of evidence-based clinical guidelines [17, 54, 55]. Changes in the childbirth model of care, aiming at a more positive experience for women, are fundamental for future choices, due to the importance of previous experiences when choosing the type of birth in new pregnancies [17, 25, 41, 42, 46]. In this study, the experiences of previous births were the second reason most cited by women who preferred a vaginal birth and the first reason for those who preferred a cesarean section.

This study has some limitations. We collected data on women´s preference retrospectively, after childbirth had occurred, which may have affected the reporting of women’s preference during pregnancy. Studies with a prospective design are recommended to measure women´s preference and changes in preference throughout pregnancy [30]. Data related to activities directed at women are not available for the period prior to the implementation of the PPA, which prevents assessing whether there was an increase in the activities offered. We also did not investigate the reasons for the low implementation of these activities. Women participating in the PPA and in the standard of care model where assisted in the same hospitals and we cannot assure that contamination did not occur. Therefore, differences in the implementation of activities directed at women may be underestimated. The external validity of the results is limited, as the hospitals included in this study are a convenience sample of Brazilian private hospitals that participated in the PPA. However, there is theoretical plausibility for the observed effects of the program on the preference for vaginal birth at the end of pregnancy, and it is possible that similar effects are observed in other hospitals in the private sector.

## Conclusion

The results of this study demonstrate the positive effects of the quality improvement project “Adequate Childbirth Project” on women’s preference for vaginal birth at the end of pregnancy. The implementation of activities directed at women was unsatisfactory and it is plausible that more intense effects will be observed with the expansion of this implementation. Special attention should be given to information and educational activities on the benefits of vaginal birth in early pregnancy, as the preference for this type of birth at the beginning of pregnancy was the main predictor of preference for VB at the end of pregnancy. The identification of barriers and facilitators for the implementation of the planned activities is necessary. Quality improvement projects that increase the participation of women in the decision-making process are fundamental for the implementation of childbirth models of care that consider the needs of women.

### Supplementary Information

**Supplementary Material 1. **

## Data Availability

The datasets used during the current study are available at Leal, Maria do Carmo (Coord.), 2023, “Nascer Saudável”, 10.35078/C1PSMZ.

## Abbreviations

ANS: National Supplementary Health Agency
CS: Cesarean section
PPA: Adequate Childbirth Project
VB: Vaginal birth
VBAC: Vaginal birth after cesarean section

## References

[1] DATASUS. Sistema de Informações sobre Nascidos Vivos. Available at http://tabnet.datasus.gov.br/cgi/tabcgi.exe?sinasc/cnv/nvuf.def. Accessed 24 Feb 2022.

[2] Rebelo F, Rocha CMM, Cortes TR, Dutra CL, Kac G (2010). High cesarean prevalence in a national population-based study in Brazil: the role of private practice. Acta Obstet Gynecol Scand.

[3] Barros AJD, Santos IS, Matijasevich A, Domingues MR, Silveira M, Barros FC (2011). Patterns of deliveries in a Brazilian birth cohort: almost universal cesarean sections for the better-off. Rev Saúde Pública.

[4] Hopkins K, de Lima Amaral EF, Mourão AN (2014). The impact of payment source and hospital type on rising cesarean section rates in Brazil, 1998–2008. Birth.

[5] Boerma T, Ronsmans C, Melesse DY, Barros AJD, Barros FC, Juan L (2018). Global epidemiology of use of and disparities in caesarean sections. Lancet.

[6] Gama SG, Viellas EF, Torres JA, Bastos MH, Brüggemann OM, Theme Filha MM (2016). Labor and birth care by nurse with midwifery skills in Brazil. Reprod Health.

[7] Leal MC, Pereira APE, Domingues RMSM, Theme-Filha MM, Dias MAB, Nakamura-Pereira M (2014). Obstetric interventions during labor and childbirth in Brazilian low-risk women. Cad Saude Publica.

[8] Torres JA, Domingues RMSM, Sandall J, Hartz ZMA, Gama SGN, Theme-Filha MM (2014). Caesarean section and neonatal outcomes in private hospitals in Brazil: comparative study of two different perinatal models of care. Cad Saude Publica.

[9] Mazzoni A, Althabe F, Liu NH, Bonotti AM, Gibbons L, Sánchez AJ (2011). Women’s preference for caesarean section: a systematic review and meta-analysis of observational studies. BJOG.

[10] Domingues RMSM, Dias MAB, Nakamura-Pereira M, Torres JA, D´Orsi E, Pereira APE (2014). Process of decision-making regarding the mode of birth in Brazil: from the initial preference of women to the final mode of birth. Cad Saude Publica.

[11] Potter EJ, Berquó E, Perpetuo IHO, Leal OF, Hopkins K, Souza MR (2001). Unwanted caesarean sections among public and private patients in Brazil: prospective study. BMJ.

[12] Agência Nacional de Saúde Suplementar. Parto Adequado. http://www.ans.gov.br/gestao-em-saude/parto-adequado. Accessed 30 Nov 2020.

[13] Borem P, de Cássia SR, Torres J, Delgado P, Petenate AJ, Peres D (2020). A quality improvement initiative to increase the frequency of vaginal delivery in Brazilian Hospitals. Obstet Gynecol.

[14] Haines HM, Rubertsson C, Pallant JF, Hildingsson I (2012). The influence of women’s fear, attitudes and beliefs of childbirth on mode and experience of birth. BMC Pregnancy Childbirth.

[15] Rosenberg KR, Trevathan WR (2018). Evolutionary perspectives on cesarean section. Evol Med Public Health.

[16] WHO recommendations: intrapartum care for a positive childbirth experience. Geneva: World Health Organization; 2018. Available at https://apps.who.int/iris/bitstream/handle/10665/260178/9789241550215-eng.pdf;jsessionid=20CAEFFFC796ADA53356CA593C703DA2?sequence=1. Accessed 27 Nov 2020. 30070803

[17] Betrán AP, Temmerman M, Kingdon C, Mohiddin A, Opiyo N, Torloni MR (2018). Interventions to reduce unnecessary caesarean sections in healthy women and babies. Lancet.

[18] Chaillet N, Dumont A (2007). Evidence-based strategies for reducing cesarean section rates: a meta-analysis. Birth.

[19] Borem P, Ferreira JBB, da Silva UJ, Valério Júnior J, Orlanda CMB (2015). Increasing the percentage of vaginal birth in the private sector in Brazil through the redesign of care model. Rev Bras Ginecol Obstet.

[20] Robson MS (2001). Can we reduce the caesarean section rate?. Best Pract Res Clin Obstet Gynaecol.

[21] Torres JA, Leal MDC, Domingues RMSM, Esteves-Pereira AP, Nakano AR, Gomes ML (2018). Evaluation of a quality improvement intervention for labour and birth care in Brazilian private hospitals: a protocol. Reprod Health.

[22] Associação Brasileira de Empresas de Pesquisa. Critério Brasil de classificação econômica. http://www.abep.org/criterio-brasil. Accessed 25 May 2020.

[23] Textor J, Zander B, Gilthorpe MS, Liskiewicz M, Ellison GT (2016). Robust causal inference using directed acyclic graphs: the R package‘dagitty’. Int J Epidemiol.

[24] Stoll KH, Hauck YL, Downe S, Payne D, Hall WA, International Childbirth Attitudes- Prior to Pregnancy (ICAPP) Study Team (2017). Preference for cesarean section in Young nulligravid women in eight OECD countries and implications for reproducitve health education. Reprod Health.

[25] O’Donovan C, O’Donovan J (2018). Why do women request an elective cesarean delivery for non-medical reasons? A systematic review of the qualitative literature. Birth.

[26] Fuglenes D, Aas E, Botten G, Oian P, Kristiansen IS (2011). Why do some pregnant women prefer cesarean? The influence of parity, delivery experiences, and fear. Am J Obstet Gynecol.

[27] Torloni MR, Betran AP, Montilla P (2013). Do Italian women prefer cesarean section? Results from a survey on mode of delivery preferences. BMC Pregnancy Childbirth.

[28] Mazzoni A, Althabe F, Gutierrez L, Gibbons L, Liu NH, Bonotti AM (2016). Women’s preferences and mode of delivery in public and private hospitals: a prospective cohort study. BMC Pregnancy Childbirth.

[29] Long Q, Kingdon C, Yang F, Renecle MD, Jahanfar S, Bohren M (2018). Prevalence of and reasons for women’s, family members’, and health professionals’ preferences for cesarean section in China: a mixed-methods systematic review. PloS Med.

[30] Schantz C, Loenzien M, Goyet S, Ravit M, Dancoisne A, Dumont A (2019). How is women’s demand for caesarean section measured? A systematic literature review. PLoS One.

[31] Dias MAB, Domingues RMSM, Pereira AP, Fonseca SC, Gama SGN, Theme-Filha MM (2008). Trajetória das mulheres na definição pelo parto cesáreo: estudo de caso em duas unidades do sistema de saúde suplementar do estado do Rio de Janeiro. Ciênc Saúde Coletiva.

[32] Hopkins K (2000). Are Brazilian women really choosing to deliver by cesarean?. Soc Sci Med.

[33] Osis MJD, Pádua KS, Duarte GA, Souza TR, Faúndes A (2001). The opinion of Brazilian women regarding vaginal labor and cesarean section. Int J Gynaecol Obstet.

[34] Barbosa GP, Giffin K, Ângulo-Tuesta A, Gama AS, Chor D, D’Orsi E (2003). Parto cesáreo: quem o deseja? Em quais circunstâncias?. Cad Saúde Pública.

[35] Weaver JJ, Statham H, Richards M (2007). Are there “unnecessary” cesarean sections? Perceptions of women and obstetricians about cesarean section for nonclinical indications. Birth.

[36] Hug I, Chattopadhyay C, Mitra GR, Kar Mahapatra RM, Schneider MC (2008). Maternal expectations and birth-related experiences: a survey of pregnant women of mixed parity from Calcutta. India Int J Obstet Anesth.

[37] Dweik D, Girasek E, Toreki A, Meszaros G, Pal A (2014). Women’s antenatal preferences for delivery route in a setting with high cesarean section rates and a medically dominated maternity system. Acta Obstet Gynecol Scand.

[38] Kingdon C, Downe S, Betran AP (2018). Women’s and communities’ views of targeted educational interventions to reduce unnecessary caesarean section: a qualitative evidence synthesis. Reprod Health.

[39] Ryding EL, Lukasse M, Parys AS (2015). Fear of childbirth and risk of cesarean delivery: a cohort study in six European countries. Birth.

[40] Colomar M, Opiyo N, Kingdon C, Long Q, Nion S, Bohren MA (2021). Do women prefer caesarean sections? A qualitative evidence synthesis of their views and experiences. PLoS ONE.

[41] Toohill J, Fenwick J, Gamble J, Creedy DK (2014). Prevalence of childbirth fear in Australian sample of pregnant women. BMC Pregnancy Childbirth.

[42] Nilsson C, Lundgren I, Karlström A, Hildingsson I (2012). Self-reported fear of childbirth and its association with women´s birth experience and mode of delivery: a longitudinal population-based study. Women Birth.

[43] Mascarello KC, Matijasevich A, Barros AJD, Santos IS, Zandonade E, Silveira MF (2017). Repeat cesarean section in subsequent gestation of women from a birth cohort in Brazil. Reprod Health.

[44] Nakamura-Pereira M, do Carmo Leal M, Esteves-Pereira AP, Domingues RM, Torres JA, Dias MA (2016). Use of Robson classification to assess cesarean section rate in Brazil: the role of source of payment for childbirth. Reprod Health.

[45] Rudey EL, Leal MDC, Rego G (2020). Cesarean section rates in Brazil: trend analysis using the Robson classification system. Medicine (Baltimore).

[46] Ryan GA, Nicholson SM, Morrison JJ (2018). Vaginal birth after caesarean section: current status and where to from here?. Eur J Obstet Gynecol Reprod Biol.

[47] Bonzon M, Gross MM, Karch A, Grylka-Baeschlin S (2017). Deciding on the mode of birth after a previous caesarean section - an online survey investigating women’s preferences in Western Switzerland. Midwifery.

[48] Horey D, Kealy M, Davey M, Small R, Crowther CA. Interventions for supporting pregnant women's decision-making about mode of birth after a caesarean. Cochrane Database Syst Rev. 2013(7):CD010041. 10.1002/14651858.CD010041.pub2. 10.1002/14651858.CD010041.pub2 PMC11608817 23897547

[49] Nilsson C, Lundgren I, Smith V, Vehvilainen-Julkunen K, Nicoletti J, Devane D (2015). Women-centred interventions to increase vaginal birth after caesarean section (VBAC): a systematic review. Midwifery.

[50] Lipschuetz M, Guedalia J, Rottenstreich A, Novoselsky Persky M, Cohen SM, Kabiri D (2020). Prediction of vaginal birth after cesarean deliveries using machine learning. Am J Obstet Gynecol.

[51] Attanasio L, Kozhimannil KB, Kjerulff KH (2019). Women’s preference for vaginal birth after a first delivery by cesarean. Birth.

[52] Fobelets M, Beeckman K, Buyl R, Healy P, Grylka-Baeschlin S, Nicoletti J (2019). Preference of birth mode and postnatal health related quality of life after one previous caesarean section in three European countries. Midwifery.

[53] Leal MC, Bittencourt AS, Esteves-Pereira AP, Ayres BVS, Silva LBRA, Thomaz EBAF (2019). Progress in childbirth care in Brazil: preliminary results of two evaluation studies. Cad Saude Publica.

[54] Oliveira CF, Ribeiro AAV, Luquine CD, Bortoli MC, Toma TS, Chapman EMG, Barreto JOM (2020). Barriers to implementing guideline recommendations to improve childbirth care: rapid review of evidence. Rev Panam Salud Publica.

[55] Barreto JOM, Bortoli MC, Luquine CD, Oliveira CF, Toma TS, Ribeiro AAV (2020). Implementation of the National Childbirth Guidelines in Brazil: barriers and strategies. Rev Panam Salud Publica.

