# Supplementary material for: Women’s preference for a vaginal birth in Brazilian private hospitals: effects of a quality improvement project
Source: Reprod Health. 2024 Mar 28;20(Suppl 2):188. doi: 10.1186/s12978-024-01771-8 (PMC10976663; doi:10.1186/s12978-024-01771-8)

Figure 1: Causal diagram for preference of type of birth at the end of pregnancy in primiparous women

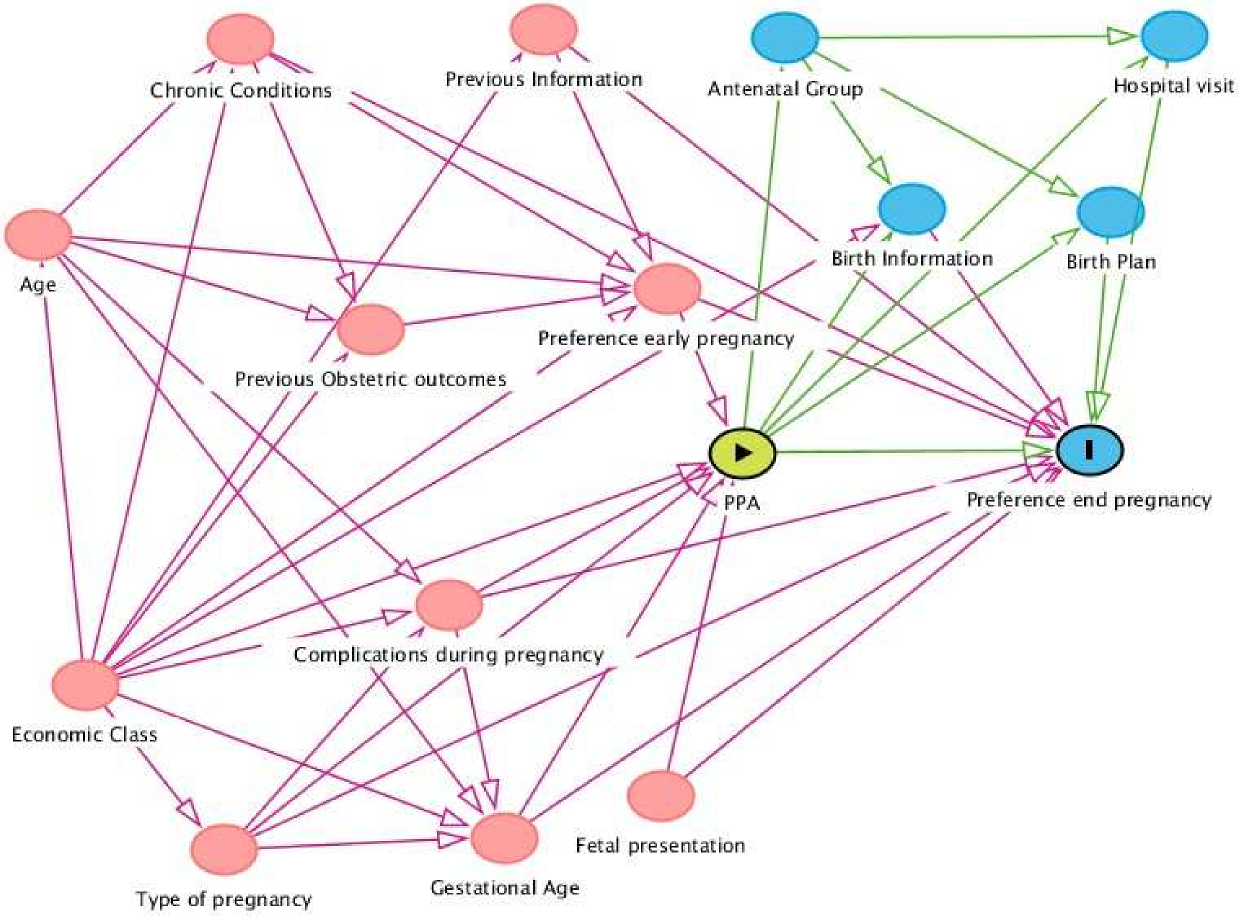

Figure 2: Causal diagram for preference of type of birth at the end of pregnancy in multiparous women

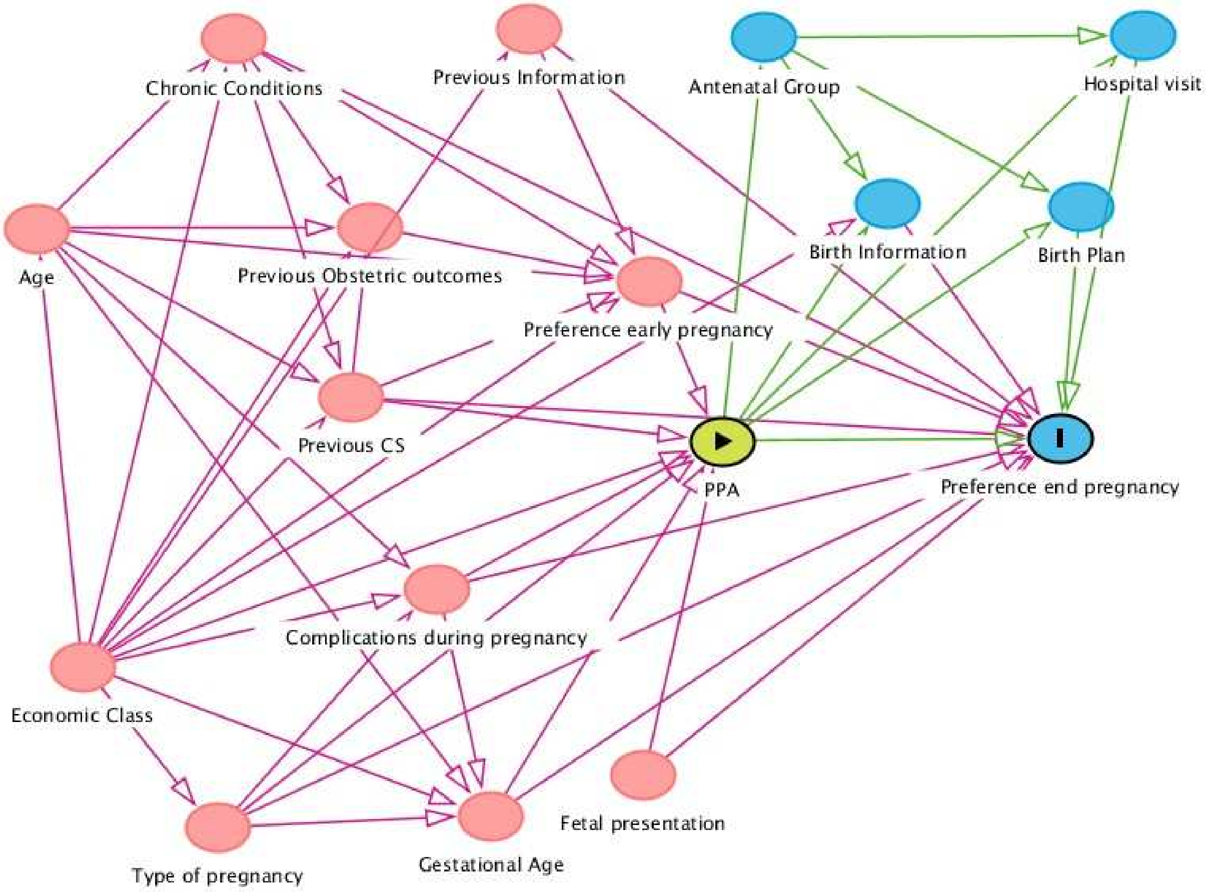

Supplement: Supplementary file 1 — Supplementary Material 1. [file 12978_2024_1771_MOESM1_ESM.pdf]
